# Supplementary figures and images for: Interactions between calmodulin and neurogranin govern the dynamics of CaMKII as a leaky integrator
Source: PLoS Comput Biol. 2020 Jul 17;16(7):e1008015. doi: 10.1371/journal.pcbi.1008015 (PMC7390456; doi:10.1371/journal.pcbi.1008015)

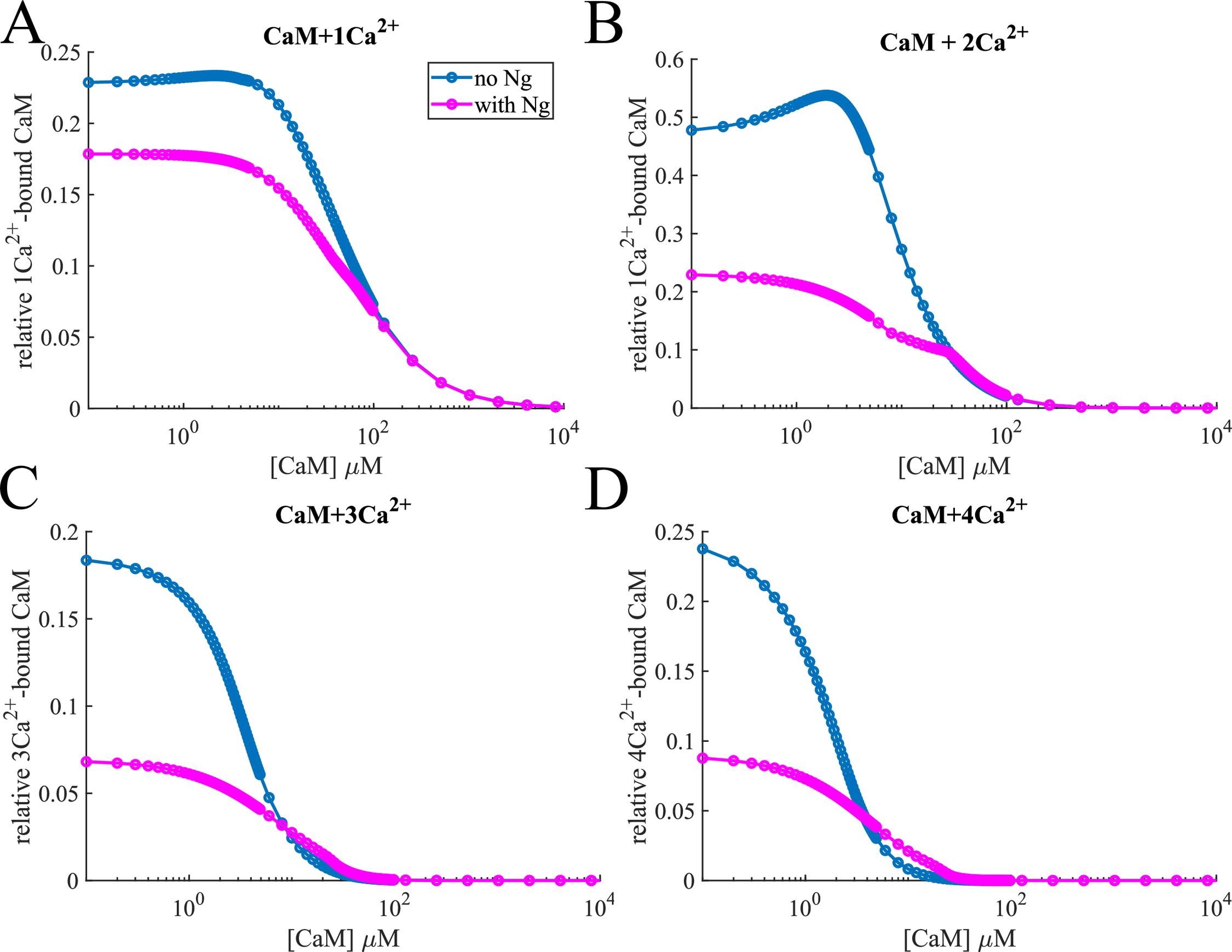

Supplement: S1 Fig — Relative distribusion of Ca2+-bound CaM species in the absence of CaMKII. (TIF) [file pcbi.1008015.s001.tif]

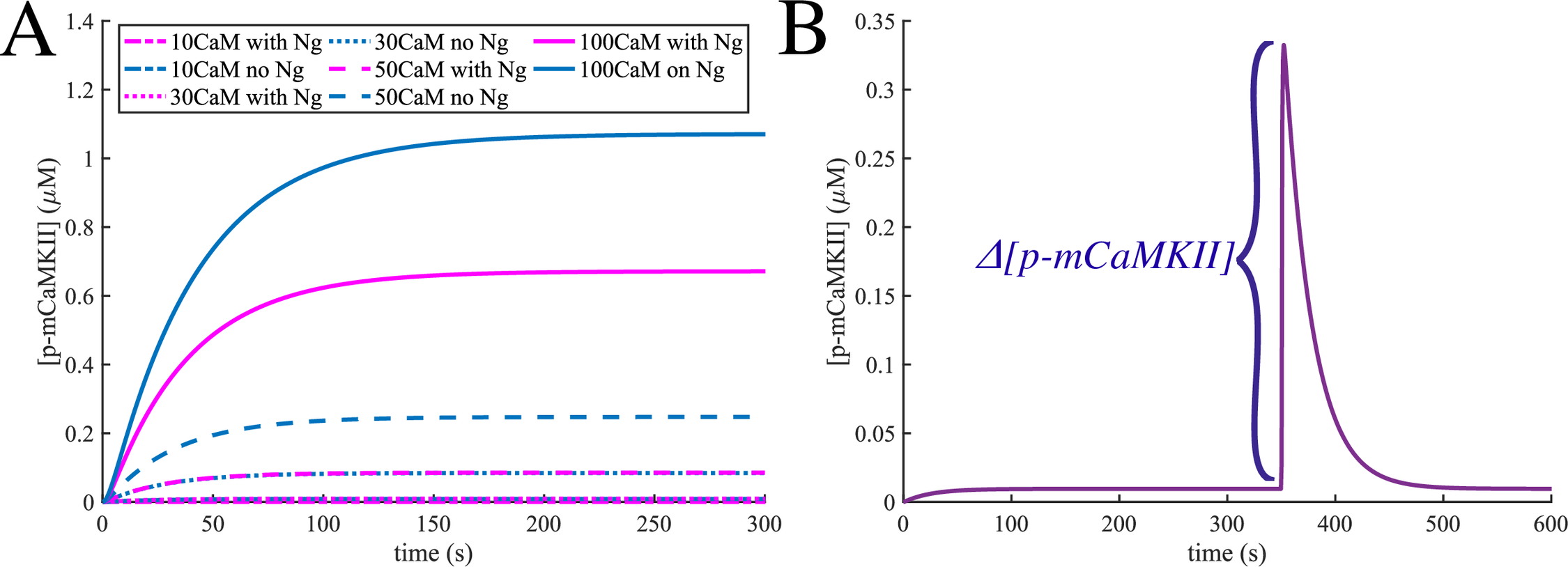

Supplement: S2 Fig — (A) at 100 nM [Ca2+] different concentrations of CaM result in different baseline levels of mCaMKII phosphorylation. (B) to enable comparisons between different conditions we compare the increase in mCaMKII phosphorylation levels (Δ[p-mCaMKII]) as shown. (TIF) [file pcbi.1008015.s002.tif]

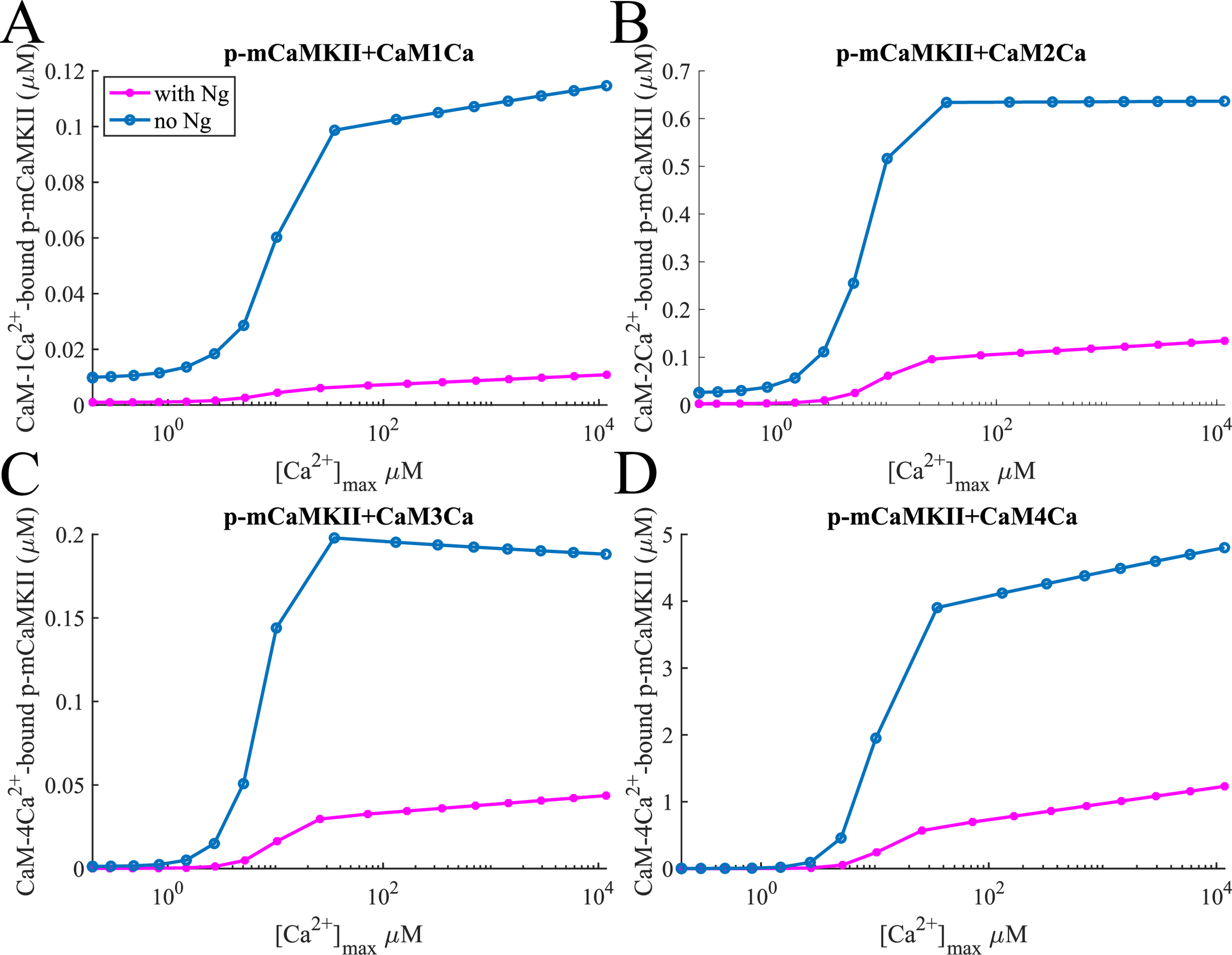

Supplement: S3 Fig — Dose response to Ca2+ spikes factored into p-mCaMKII bound to different species of Ca2+/CaM. (TIF) [file pcbi.1008015.s003.tif]

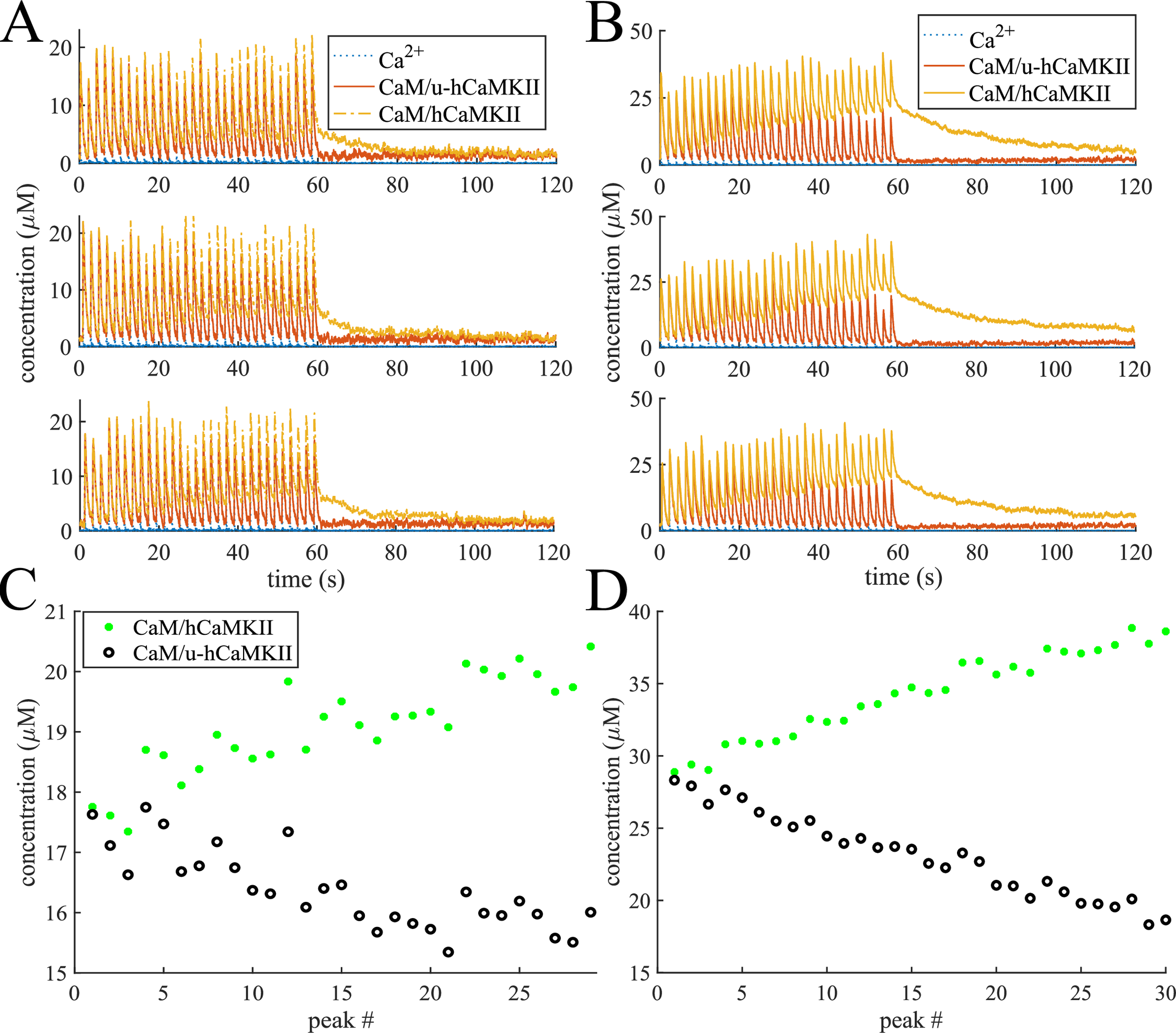

Supplement: S4 Fig — Comparing total and unphosphorylated hCaMKII (u-hCaMKII) bound to CaM with (A) and without (B) Ng: while total CaM-bound hCaMKII decays slower after a Ca2+ spike, CaM-bound u-hCaMKII gets down to base level immediately after the spike. C and D show the 30 peaks reached by CaM-bound hCaMKII and u-hCaMKII averaged over 30 simulations, with and without Ng respectively. (TIF) [file pcbi.1008015.s004.tif]

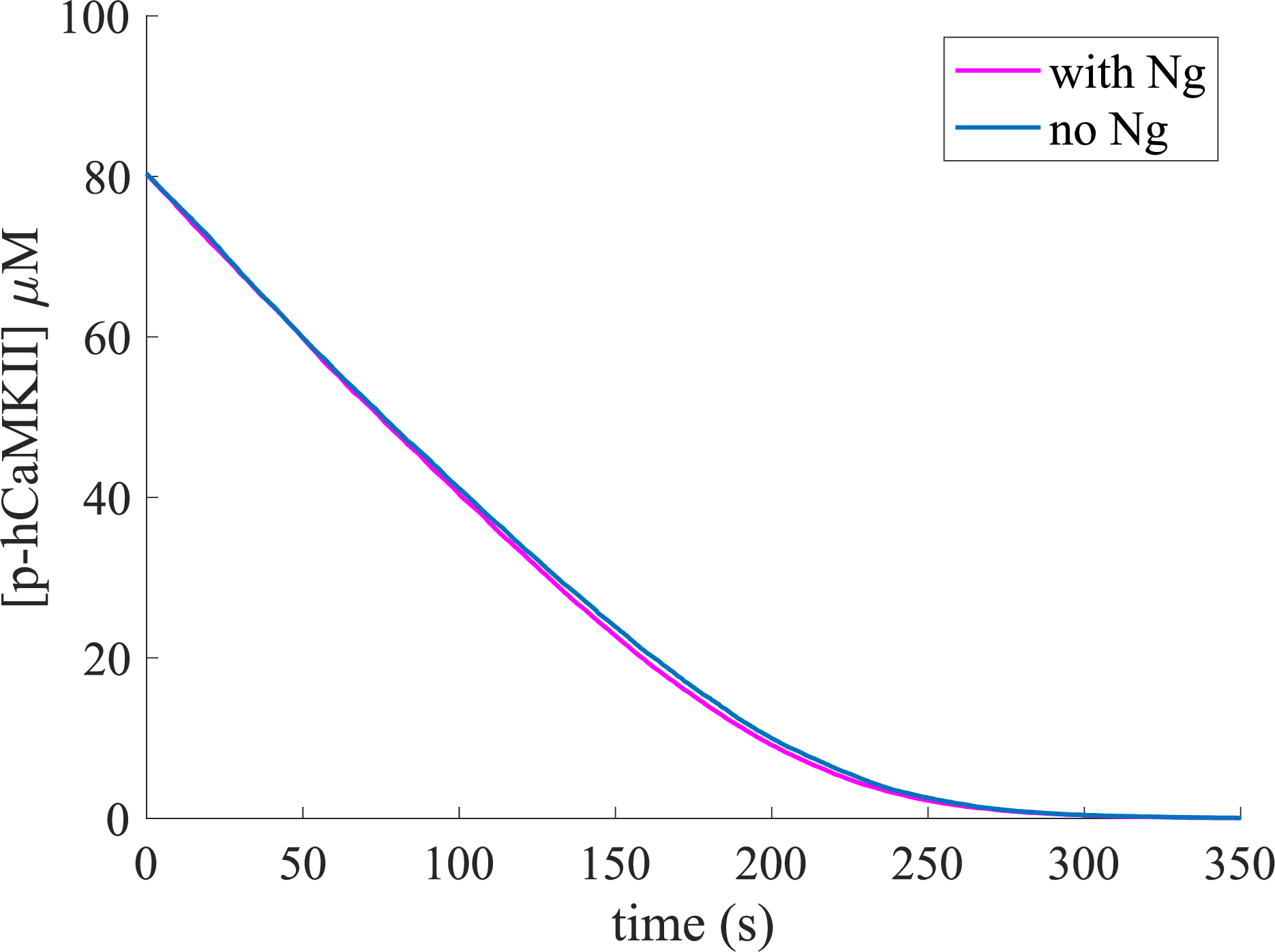

Supplement: S5 Fig — hCaMKII does not exhibit bistability at [Ca2+] = 100nM in our model: even when all of hCaMKII is phosphorylated, the activation decays back to base level. (TIF) [file pcbi.1008015.s005.tif]
