# Supplementary material for: Interactions between calmodulin and neurogranin govern the dynamics of CaMKII as a leaky integrator
Source: PLoS Comput Biol. 2020 Jul 17;16(7):e1008015. doi: 10.1371/journal.pcbi.1008015 (PMC7390456; doi:10.1371/journal.pcbi.1008015)
Supplement: Attachment — Submitted filename: Responses_Final.pdf [file pcbi.1008015.s006.pdf]

Response to Reviewers:

We thank the reviewers for their extensive, diligent, and insightful comments. We have responded to specific concerns below and modified the manuscript accordingly; changes in the manuscript are highlighted in blue in a separate PDF file attached for review only. The reviewer's comments are given in **bold face** with our response in plain text. Where appropriate, portions of the manuscript that are changed are provided in red.

Reviewer 1:

We thank the reviewer for their comments and for acknowledging the importance of the presented work. Below are the responses to their comments.

1.1. Previously several different models of CaMKII activation in spines have been developed. One of the most relevant ones would be a model developed by Zhabotinsky et al (2002, J. Neurosci), as it includes all molecular components discussed in the current paper, including CaM, CaMKII, Neurogranin and phosphatase. The authors should go through previous models of CaM-neurogranin binding and its role in CaMKII activation in the introduction and discussion. In particular, the paper would be enhanced by more explicit explanation of the difference from previous attempts and benefits of the presented model.

We thank the reviewer for drawing our attention to previous attempts of CaMKII modeling. The reviewer is correct that previous models have considered these interactions and we have now briefly mentioned it in our paper. We also note that to our knowledge, these models did not consider the interactions between CaM and monomeric CaMKII. We have added the following sentences to the introduction of our paper to clarify the differences between our model and objectives, and those pursued by others in the field (page 4, last paragraph):

"Many computational models of CaMKII dynamics have been developed in the literature to probe the different interactions in this cascade at varying levels of detail (Lisman et al 1985, Zhabotinsky et al 2006, Zhabotinsky et al 2000, Lisman et al 2012, Lisman et al 2002, Lisman et al 2001, Graupner et al 2007, Pharris et al 2019, Okamoto et al 2000). A large majority of these models focused on bistability behavior of CaMKII (Lisman et al 1985, Lisman et al 2002, Lisman et al 2001, Zhabotinsky et al 2000,Graupner et al 2007, Okamoto et al 2000), which were obtained by the nonlinear rate functions used in the model. However, experiments indicate that in long-term potentiation, CaMKII is only transiently activated and does not exhibit bistable behavior Lengyel et al 2004, Lee et al 2009. Furthermore, as noted recently (Michalski 2014), CaMKII is a much more complicated molecule because of its multimeric nature and the intersubunit interaction for the T286 phosphorylation, which are affected by Ng and by phosphatases. Therefore, a computational model for probing CaMKII dynamics needs to account for both monomer behavior and

therefore the resulting holoenzyme dynamics of CaMKII in the presence of Ng and PP1."

1.2. Dephosphorylation rate of bulk CaMKII in spines was measured to be 6 s (Lee et al., 2009 Nature; Chang et al., 2017 Neuron, Chang et al., 2019; Nat Commu) at room temperature and perhaps much faster in physiological temperature, instead of 60 s used in the simulation. The author could comment on the impact of this on the simulation.

We thank the reviewer for pointing out the difference between the results of our simulation and the experimental observations. We would like to point out that the 60s time it took for the CaMKII to come back to its basic levels in our model is a result of the Michaelis-Menten constants of CaMKII dephosphorylation by PP1. The rate constants for this particular reaction were measured by Bradshaw et al 2003. We have added the following sentences to our manuscript to compare these different parameters (page 8, last paragraph):

"We note that our simulations result in phosphorylation lifetime on the order of minutes. This is longer than the time constants of 6 and 45 seconds measured by [41]. This discrepancy may be because the dephosphorylation time constants measured in spines might be a result of the spatial organization of the spine, not taken into account here, or additional molecular pathways that are not a part of the current model."

1.3. The paper would benefit from additional discussion about the kinetics of CaM-CaMKII binding in the simulation compared with that measured in spines (Chang et al., 2019, Nat Commu).

We thank the reviewer for this suggestion. We have now added a new figure (Figure 10) and a supplemental figure (Figure S4) to compare our modeling results with the experimental results from Chang et al. 2019. We also added the following paragraph to the manuscript (page 17, last paragraph)

"We also found that hCaMKII activation through Ca^{2+}/CaM binding does not integrate over calcium signals the way hCaMKII phosphorylation does. Figure 10 shows 3 representative examples of the changes of CaM-bound hCaMKII concentration in response to Ca^{2+} spikes at $CaM = 50\mu M$ and in the presence and absence of Ng. This finding is consistent with recent observations in Chang et al. 2019. We observe that when Ng is present (Figure 10A) hCaMKII activation through CaM binding reaches about the same level at every Ca^{2+} spike. This is what was observed experimentally by Chang at al. 2019. On the other hand, the subsequent decrease of CaM-bound-hCaMKII after each pulse stops at higher concentrations after later Ca^{2+} spikes. This is particularly evident in the absence of Ng (Figure 10B). In fact, in this case, we can also see a slight increase in peak levels of CaM-bound hCaMKII. When we look at only unphosphorylated hCaMKII molecules (u-hCaMKII) bound to CaM; however, we see that the peaks of these curves slightly decrease, while the troughs remain at the same level (Figure S4A and B). This prediction was also observed experimentally with T286A CaMKII mutation, that cannot be phosphorylated Chang et al. 2019. Finally, when we look at the average peak concentration of CaM bound hCaMKII and u-hCaMKII corresponding to

each Ca^{2+} pulse over 30 trials we see that on average peaks corresponding to later pulses are slightly higher for CaM/hCaMKII and slightly lower for CaM/u-hCaMKII species (Figure S4 C and D). It is noteworthy that, while Chang et al. observed no increase in CaM/CaMKII peak concentration with later glutamate uncaging pulses, they also found that phosphorylation at T305 site accelerates CaMKII inactivation. The fact that our model does not include this phosphorylation site, might be the reason we see an average increase in peak CaM-bound hCaMKII concentration."

1.4. Since Neurogranin is phosphorylated by PKC, which is activated by Ca2+ influx into spines (Colgan et al., 2018, Nat Neuro), and releases CaM as suggested by Zhabotinsky et al. (2002, J. Neurosci). Discussion on potential impact to their simulation would enhance the paper.

We thank the reviewer for this comment. We agree that the phosphorylation of Ng by PKC is an important part of the puzzle and needs a careful consideration. As we note in the revised discussion, a detailed model of this pathway is something we are interested in, and are working to develop. It is, however, outside the scope of the current work. We have now added the following sentences to the manuscript to address this (page 23 paragraph 2):

"An additional detail, not considered in the present work, is the modulation of Ng activity through protein kinase C (PKC). Ca^{2+} influx in spines activates PKC, which phosphorylates Ng. Phosphorylated Ng has diminished ability to bind CaM (Diez et al 2010, Colgan et al. 2018), resulting in altered dynamics of CaMKII phosphorylation and further fine-tuning of the synaptic response to stimulation. Integrating the detailed pathway of PKC-Ng interaction is the focus of a future study."

Reviewer 2:

The manuscript by Ordyan et al addresses an important question around how CaMKII activation is controlled in spines. The model is based on earlier models and is an as solid model as one could wish for to make predictions and increase our understanding for the dynamic signaling underlying CamKII dependent plasticity in spines. The simulations are testing the role of Cam, Ng and Ca for the activation of CaMKII (incl phosphorylation). Several important predictions are made, and to increase the understanding from the readers point of view, additional explanations will be useful. Since the model is not that big and one can measure all variables in parallel and manipulate amounts, kinetics, etc, one should also be able to explain the mechanisms in the model that the results depend on. Thus ideally the results are not only model predictions, but explainable predictions given the current model. I have listed questions I asked myself when reading the manuscript. If these items can be explained in even more intuitive ways, this work will make a much larger impact.

We thank the reviewer for their positive assessment of our work and their critical review aimed to make the work more impactful. We have now extensively revised the manuscript with an eye towards addressing these points and hope that the reviewer finds our modifications as a significant improvement to the manuscript. Below are our responses to each comment.

2.1 Explain better (e.g. on p6 or in methods) how the Ca input was modelled. And maybe show the input signal (it is shown in fig 8, but would be good to see one of the 10mikroM transients used for the first few figs in a clearer way). Was the Ca_free level clamped to a certain level, e.g. during the 10mikroM transients, such that it didnt matter how much Cam was around? Thus much more Ca was bound to Cam in those simulations where the Cam amount was higher (thus assuming that more calcium was sequestered in the PSD)? If one instead had injected a certain amount of Ca for each input (and assumed a removal machinery for the Ca such that it went down to resting state within 100ms or so), how would that have affected the results? Then perhaps one shouldnt necessarily see an increased CamKII activation for more Cam. Please discuss (or test) these things and relate to a reasonable scenario for real spines. Perhaps one could speculate that if there were more Cam it also requires higher Ca influx for efficient CamKII activation? Is also calcium limiting if Cam is there in high amounts, and vice versa?

We apologize for the confusion our wording might have caused regarding the Ca^{2+} signal in the earlier part of the paper. In our initial simulations (Figures 2-4) total calcium concentration is set to be 10 µM throughout the simulation. We have now added the phrase "a constant calcium concentration of $[Ca^{2+}] = 10$ µM" to the first paragraph of the Results (page 4) section to make this clearer. Figure 3 presents final CaMKII phoshporylation levels at constant total calcium (10µM) at different [CaM]s. Figure 4A on the other hand, presents the response of the system containing 30µM CaM to different calcium signals. We have now extensively modified the figure captions and text to reflect these points clearly.

2.2 On p7 it is said that Interestingly, the phosphorylation dynamics with Ng and a 50 mikroM Cam is identical to that without Ng at 30 miroM Cam. Can this be explained? Is this because 20 mikroM Ng binds almost 20 mikroM Cam, thus 30mikroM available to bind to Ca? But then, why is this not the case for the holoenzyme in Fig 6B?

We thank the reviewer for pointing out this important point. Indeed, the reason for the identical dynamics is that almost 20 µM CaM is bound to Ng. This effect is not apparent in the case of the holoenzyme in Fig 6B because we show the average response of 30 different simulations. This becomes clear when comparing the error bars presented on Fig. 7. To clarify this point we have added inset plots of Ng-bound CaM to Fig. 6 A and B as suggested in point 2.8. We have also added the following passages to the section discussing results on Fig 6A (page 8, paragraph 2):

"This similarity can be easily understood, by looking at the concentrations of Ng-bound CaM (CaM.Ng) at

different [CaM]s. As shown in the inset of Figure 6A, when $[CaM] = 10\mu M$ nearly all of it is bound to Ng, and when $[CaM] > 20\mu M$ approximately 20µM of it is bound to Ng."

And the following passage was added to the section discussing the analogous holoenzyme results on Fig. 6B and Fig. 7 (page 15, last paragraph):

"In the inset of Figure 6B we show again that when $[CaM] > 20\mu M$ nearly 20 µM of it is bound to Ng. In this light, it might be surprising that the response curves for [CaM] = 30, and 50 µM do not overlap as nicely as they do in Figure 6A. This is because the individual simulations are stochastic; comparing the results on Figures 7B, D, F and H we can see that the average results of 30 simulations for [CaM] = 30 µM without Ng, and [CaM] = 50 µM with Ng are consistent with each other within the error bars presented."

2.3 In Fig 2, the presence of Ng can allow a higher relative Cam4Ca amount. This is explained with the Ca/Cam ratio. Is this behavior also seen if one assumes the CamKII amount is zero? I.e. is this behaviour a result of only competition between Ca and Ng for Cam, or does CamKII play a role as a leaky buffer for the multi-calcium-bound forms?

We thank the reviewer for pointing out this possibility. We conducted the necessary simulation, where we kept the interactions between and concentrations of Ca^{2+} and CaM the same, but excluded CaMKII.

The results of these simulations have been added as a supplemental figure (Figure S1). We also added the following sentences to the manuscript (page 6 paragraph 2):

"These observations lead to an interesting question: is the higher relative concentration of $4Ca^{2+}/CaM$ resulting truly from the competition between calcium and Ng for CaM or does CaMKII play a role as a leaky buffer for different multi-calcium-bound forms? To answer this question, we repeated the simulations in the absence of any CaMKII. In this case, we observed an even more pronounced effect of Ng in the increase of multi-calcium-bound states of CaM for certain CaM concentrations (Figure S1) but the overall trend remains the same as in the presence of CaMKII (Figure 2). Thus, we conclude that the relative distribution of different calcium-bound forms of CaM are indeed a result of competition between Ng and Ca^{2+} for CaM."

2.4 In fig 4A the steady state activation of CamKII is shown for higher Cam levels. The second peak is due to Cam with few Ca bound. Is the reason that we dont see that for the transient Ca input used in fig 3 that there is not enough time for that during 100ms or so? One can perhaps also notice that for physiological levels of Cam (e.g. 10mikroM) the response reaches up to 2.5 mikroM. If one compares with fig 3 the amplitude is just around 1. It thus seems that during a Ca transient less than half of the steady-state level is reached. But in fig 4C and 4D it seems to take a long time to reach steady-state. Explain these things better in the text, please.

Again, we apologize for any confusion our wording might have caused, and we have now tried to make it clearer in the manuscript that for our initial simulations (Figures 2-4) the concentration of total calcium

was set to be constant. Since we always start with no Ca^{2+} bound to CaM, and set the $[Ca^{2+}] = 10\mu M$ in the beginning of the simulation, it takes some time to reach equilibrium as shown in Figures 4C and D, and noted by the reviewer. We hope that in light of this clarification it becomes clear that Figures 3 and 4A are illustrations of the results of the same sets of simulations. The reason that the numbers from Figure 3 do not add up to the ones shown in Figure 4A as the reviewer noticed (1 vs 2.5) is that the count of phosphorylated CaMKII on Figure 4A contains all of pCaMKII present, including the molecules that have unbound from CaM, whereas on Figure 3 we only count pCaMKII that is bound to a particular species of Ca^{2+}/CaM. We have now modified the last paragraph on page 6 to address this:

"Here, the count of p-mCaMKII includes the molecules that have released the Ca^{2+}/CaM complexes they were bound to while being phosphorylated. Thus, this count is higher than the total sum of the p-mCaMKII molecules bound to different species of Ca^{2+}/CaM plotted on Figure 3.With this information, we can interpret the results shown on Figure 4A after having examined those on Figure 3.Here, the first local maximum is caused by phosphorylation of mCaMKII bound to $4 - Ca^{2+}/CaM$. Since there are more of these species in the presence of Ng (Figure 3 D) this peak is more prominent when Ng is present in the system. The second peak is caused by phosphorylation of the mCaMKII bound to 1- and 2- Ca^{2+}/CaM. These species play an important role at higher [CaM], where the role of Ng is less important. Therefore, total p-mCaMKII increasingly overlaps as [CaM] is increased irrespective of the presence or absence of Ng."

2.5 Fig 5, how can pCamKII_max be so much higher without Ng? What is the relation between fig 5 and earlier figs? Also in earlier figs the results for 30 mikroM of Cam is shown at a Ca transient of 10 mikroM. Describe better and explain so readers can understand. Is the input changed here?

We hope that from our earlier clarifications it is now clear that Figure 5 is the first figure where a transient $[Ca^{2+}]_{free} = 10\mu M$ is the input. The reason $[pCaMKII]_{max}$ is so much higher without Ng, is because when Ng is present $\sim 20\mu M$ of CaM is bound to Ng, as we have now shown on Figure 6A following the reviewers suggestion. We hope that the changes we have made to the manuscript thus far will prevent the reader from getting confused.

2.6 Fig 6, why is the holoenzyme less activated than the monomer system? Is that because only the closest neighbours can be phosphorylated in the holoenzyme? Also explain what mechanism in the model makes the holoenzyme both peak earlier and decay more rapidly.

We thank the reviewer for advising us to elaborate on this points. We have added the following sentences to address these questions (page 16, second paragraph):

"This is understandable: while in the case of monomers any active molecule of CaMKII can bind and phosphorylate another, provided the latter is bound to Ca^{2+}/CaM, in the case of the holoenzyme a given subunit can only be phosphorylated by one (and only one) of its neighbors. Since the phosphorylation

of a given subunit in the holoenzyme is still dependent on it being bound to a $[Ca^{2+}/CaM]$ complex, this condition becomes harder to satisfy shortly after the $[Ca^{2+}]$ drops back to $\sim$ 100nM. And with the holoenzyme the cessation of phosphorylation happens more rapidly, since the conditions imposed for phosphorylation of a given subunit are more rigid. Thus, these more rigid conditions explain both the lower phosphorylation level, and the shorter timescales exhibited by the holoenzyme."

2.7 The manuscript highlights that sequestration effects are important, and that Cam becomes a limiting species. In this context one I think should mention another computational study that reached a similar conclusion that Cam is limiting for activating CamKII if one has phosphorylated RCS (ARPP-21), see Nair et al, Plos Comput Biol, 2016. Also Rakhilin et al 2004 is in line with that Cam limiting even for PP2B activation and that RCS competes with PP2B for activated Cam.

We thank the reviewer for this suggestion. We have now added the following sentence to our Discussion (page 23 paragraph 1).

"It has been observed that CaM becomes a limiting factor for CaMKII activation once regulator of calmodulin signaling (RCS) is phosphorylated, making this protein another competitor to bind CaM (Rakhilin et al 2004, Nair et al. 2016.)"

2.8 Since Cam binding to Ng is highlighted here and there, why not show an inset plot of the Ng-Cam in relevant figures (e.g. see also item b) above).

We thank the reviewer for this suggestion, and have addressed it as discussed in response to point 2.2.

2.9 Please discuss the role PKC phosphorylation of Ng might have, and relate to the figs in the manuscript. For example, if using more Cam in fig 3D or 4A it would not help much, but if one has more Ca it might change things? Or how does the model behave here?

We thank the reviewer for this comment. We agree that the phosphorylation of Ng by PKC is an important part of the puzzle and needs careful consideration. As such a detailed model of this pathway is something we are interested in, however we consider it outside the scope of this paper. We have now added the following sentences to the manuscript to address this(page 23 paragraph 2):

"An additional detail, not considered in the present work, is the modulation of Ng activity through protein kinase C (PKC). Ca^{2+} influx in spines activates PKC, which phosphorylates Ng. Phosphorylated Ng has diminished ability to bind CaM (Diez et al 2010 Colgan et al 2018), resulting in altered dynamics of CaMKII phosphorylation and further fine-tuning of the synaptic response to stimulation. Integrating the detailed pathway of PKC-Ng interaction is the focus of a future study."

2.10 In the methods section I think one should just acknowledge that rule-based modeling in this case implies that one assumes that allosteric effects are not so important. (although obviously it would be unpractical to simulate all combinations of molecular species, and one

would not have data on allosteric interaction anyway, at least not today).

We have added the following sentence to the methods section to address this comment: (page 27, paragraph 2)

"Any possible allosteric interactions are ignored."

2.11 Finally, maybe make sure fig texts are more transparent when it comes to what Ca input that was used, and also if monomers or holoenzymes were the readout. Seems monomers were used in the beginning and then holoenzymes investigated.

We have addressed this comment by adding the relevant information to the figure captions where applicable and hope the revised figure captions are now clearer.

Reviewer 3:

In this manuscript, the authors investigate the phosphorylation dynamics of CaMKII triggered through binding of calcium-associated calmodulin. The authors account for the sequestering of calmodulin through neurogranin and show that the presence of neurogranin has a profound impact on available calmodulin and consequently on CaMKII phosphorylation levels and dynamics. The results suggest neurogranin to be potentially important for regulating processes involved in synaptic long-term potentiation. The authors are the first to consider the interaction between calmodulin and neurogranin and the impact for CaMKII activation.

We thank the reviewer for their detailed and constructive suggestions. These suggestions have helped us significantly improve the manuscript. We address their comments and the corresponding changes we made to the manuscript below.

My general critic is that the study falls short in providing the insights and explanations which are the unique potential of such modeling studies. Another crucial part of biophysical modeling work is to propose feasible experiments which can support or contradict the conclusions drawn from the theoretical results and further elucidate the role of the protein interaction network. Such considerations are completely missing in the present work.

We thank the reviewer for this feedback. In the extensively revised manuscript, we have now made major changes to the introduction and the discussion, in addition to providing clarity in the results section throughout the manuscript. We have also discussed the role of the CaM-Ng interaction in the context of other pathways (for e.g. PKC) and how this will impact downstream signaling and structural plasticity. We hope the reviewer finds the revised manuscript is now suitable for publication.

3.1. It's also not clear how the steady-state results, which reveal the non-intuitive dependence

of pCaMKII on calmodulin concentration (e.g. Fig. 4), relate to the dynamic stimulation part, in which the authors use calcium transients to induce CaMKII phosphorylation (starting with Fig. 5). The facilitating effect of neurogranin in the steady-state results seems to be absent in the dynamic calcium simulations, where the sole effect of Ng appears to be reducing pCaMKII levels. Putting the presented results in relation would help reader comprehension.

We thank the reviewer for this comment. We have now extensively edited to manuscript to clarify the relationship between the different stimuli and different models. Indeed, as the reviewer points out, in the cases when calcium spikes are investigated the facilitating effect of Ng seems to be absent at all $[CaM]$s. However, our steady state interactions give insight into the relative distributions of calcium-bound CaMs at different levels of $[CaM]$s setting the stage for a more complete understanding of the role played by Ng in this pathway. Since our goal was to build a bottom-up approach in this paper, we believe that it is important to understand the relevant behavior of our model at every step.

3.2. As the authors point out, calmodulin contains four calcium binding sites, two at the C- and two at the N-terminal domain. One hallmark of a steep calcium-dependent activation of calmodulin is that calcium binding happens in a cooperative manner in each one of these pairs (Chin D, Means A (2000) Calmodulin: a prototypical calcium sensor. Trends Cell Biol 10: 322328). It's surprising that the authors don't account for this property crucially determining how calcium activation affects downstream targets such as CaMKII. The cooperativity strongly favors CaM+2Ca and CaM-4Ca alternating the relative concentrations curves in Fig. 2 and all subsequent results.

We thank the reviewer for this comment. We would like to clarify that cooperativity is built into our model rather than being included explicitly and is reflected in the rate constants presented in Table 1 as demonstrated on the figure below.

3.3. Previous work on CaMKII activation through CaM/Ca and autophosphorylation revealed that the protein can exhibit bistability in its phosphorylation level (Zhabotinsky 2000 Biophys J; Graupner 2007 PLoS Comp Biol). In other words, for the same calcium concentration, the protein can exist in a highly or a weakly phosphorylated state and both are stable. How does this current work relate to this line of work. Does CaMKII bistability exist in the model?

We thank the reviewer for this question. We would like to make the following clarifications about the notion of CaMKII bistability as presented in the Zhabotinsky 2000 model and point out the following sentence from Zhabotinsky 2000 Biophys J

"Fig. 3 demonstrates that e_k must exceed 10 µM, and K_M must be significantly lower than 1 µM to obtain a bistability range that includes the resting value of the intracellular Ca^{2+} and is wide enough to prevent induction of LTP by random fluctuations of Ca^{2+} concentration."

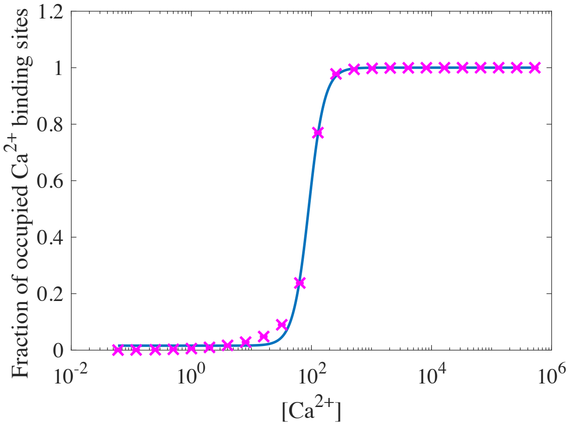

Here the pink crosses are the result of the simulated dose response of CaM to Ca^{2+}, where we have included only the reactions between CaM and Ca^{2+}. The blue line is a fitted Hill equation with a Hill coefficient $n = 3.3257$ and $EC_{50} = 90.4801$

Here e_k refers to [CaMKII], and K_M refers to the Michaelis constant of PP1. The major difference between our model and the one presented in Zhabotinsky 2000 is the source of kinetic parameters. In our model, we used an experimentally measured value of 11 µM for K_M (Table 1, Bradshaw et al 2003, PNAS) and therefore, we would not expect to see bistability. We have now added a new supplementary figure (Figure S4) and the following paragraph to our manuscript (page 23 last paragraph) to provide better context for our model in light of the bistability argument.

"Given the wealth of models for CaMKII phosphorylation dynamics in the literature, it is important to consider our results in the context of previous findings. It has been suggested by previous models that CaMKII can exhibit bistability and act as a molecular switch (Graupner et al. 2007, Zhabotinsky et al. 2000, Lisman et al. 2001). While the idea of CaMKII bistability is an appealing explanation behind the LTP phenomenon, it has not been without controversy. For example, one experimental demonstration of CaMKII activation hysteresis was performed *in vitro* at higher than physiological $[Ca^{2+}]$ $(> 200nM)$ in the presence of NMDA receptors and in a purified system (Urakubo et al. 2014). To date, to the best of our knowledge the has been no *in vivo* experimental results suggesting evidence for CaMKII bistability. Furthermore, the computational models that predict bistability at physiological Ca^{2+} concentrations require a lower Michaelis-Menten constant of CaMKII dephosphorylation by PP1 $(< 1\mu M)$ than measured experimentally (11µM, Table 1). A stochastic model of CaMKII activation by Michalski showed that CaMKII does not exhibit bistability in physiological $[Ca^{2+}]$ (Michalski 2013) and posited that the previous reports of bistability were perhaps the result of approximations of CaMKII dynamics to overcome the combinatorial explosion of multistate, multisubunit dynamics. Since our model accounts for the subunit level phosphorylation of CaMKII at the holoenzyme level (similar to Michalski), we did not find regimes of bistability with our model. Indeed, even if we initiated the simulation with all of hCaMKII in the model being phosphorylated,

the activation decays back to basic level at physiological $[Ca^{2+}] = 100nM$ (Figure S4)."

3.4. What is the rational behind depicting relative concentration for ca-bound calmodulin in Fig. 2?

Calcium and Ng compete for CaM. By demonstrating how the number of calcium ions bound to CaM in the presence and absence of Ng alters the relative concentration of calcium-bound CaM, we set the stage for understanding how mCaMKII phosphorylation is affected by the presence or absence of Ng. We hope that it can be appreciated that the results depicted on Figure 2 help understand results presented later in the paper (in particular on Figures 3 and 4). Since we have adopted a bottom-up approach in this work we think it important to be able to understand the mechanisms at work at every level of building of the model presented here.

3.5. Fig. 3 and Fig. 4 : Why is the concentration of calcium-bound calmodulin bound to phosporylated CaMKII (Fig. 3) and phosphorylated CaMKII (Fig. 4) decreasing with increasing calmodulin concentration? Even though the relative free calcium-bound calmodulin decreases with more CaM, the absolute concentration of calcium-bound calmodulin should increase or saturate. I would expect monotonously increasing concentration levels in Fig. 3 and 4.

As the reviewer mentions the relative calcium-bound calmodulin concentration decreases with more CaM. Since calcium-free CaM can still bind CaMKII, albeit with a low affinity, at higher relative concentration these species present a serious competition with underrepresented forms of calcium-bound CaM, eventually out-competing these forms at ultra-high concentrations, and resulting in lower and lower CaMKII phosphorylation.

3.6. pg. 4, 3rd paragraph : What is at the origin of the cross-over of the of the calcium-bound calmodulin with multiple calcium ions between curves in the presence and absence of Ng? Can the authors provide an intuition for this effect which does not exist for calmodulin bound with 2 or 3 calcium ions.

As we explain in the text (now last paragraph on page 5), the reason for this crossover is the competition between Ng and calcium for CaM: for certain CaM concentrations, when $Ca^{2+} : CaM_{free}$ is higher due to the presence of Ng, calcium can more easily bind a form of CaM that is already bound to calcium, since CaM_{free} is unavailable. This results in higher concentrations of multi-calcium-bound forms of CaM. We have now added a supplementary figure (Figure S1) and additional explanation. When we eliminate the interactions of CaM with CaMKII, we show that this effect can be understood as a result of competition between CaM and Ng. We have now slightly shortened and edited the mentioned paragraph (now last paragraph on page 5) as well as added an additional paragraph explaining the interactions in the absence of CaMKII as follows, and hope that it is less confusing (changes in red):

"Figure 2 shows the maximum amounts of each individual Ca^{2+}/CaM species relative to total CaM available for a range of [CaM]s. We observe that without Ng, we consistently have more $1Ca^{2+}/CaM$ than in the presence of Ng (Figure 2A, and B, and E top panels). This is easily understandable: Ng limits the availability of free calmodulin, and in its absence, we have more CaM available to bind calcium. On the other hand, when we look at CaM species that have multiple calcium ions bound to them, we see a crossover of the 2 curves as the total [CaM] increases. This is particularly striking for $3Ca^{2+}/CaM$ and $4Ca^{2+}/CaM$ (Figures 2C, D, and E bottom panels). For these species, limiting the available free CaM can be beneficial since this increases the ratio $[Ca^{2+}] : [CaM]_{free}$, resulting in higher number of calmodulin proteins bound to multiple calcium ions. Figure 2E shows the dose response in the physiological range of CaM concentration.

While the calculated effects may seem small, they are amplified by the fact that these multi-calcium-bound calmodulin species have a higher CaMKII binding rate, and render the bound CaMKII more susceptible to phosphorylation (Table 1). These observations lead to an interesting question: is the higher relative concentration of $4Ca^{2+}/CaM$ resulting truly from the competition between calcium and Ng for CaM or does CaMKII play a role as a leaky buffer for different multi-calcium-bound forms? To answer this question, we repeated the simulations in the absence of any CaMKII. In this case, we observed an even more pronounced effect of Ng in the increase of multi-calcium-bound states of CaM for certain CaM concentrations (Figure S1) but the overall trend remains the same as in the presence of CaMKII (Figure 2). Thus, we conclude that the relative distribution of different calcium-bound forms of CaM are indeed a result of competition between Ng and Ca^{2+} for CaM."

3.7. It would be instructive to see the dynamics of the different calmodulin forms (CaM-1Ca, CaM-2Ca,...) in response to the calcium transient, the summary of which is presented in Fig. 5 for pCaMKII. That could maybe also provide a link to the steady-state considerations discussed up to this point.

We thank the reviewer for this suggestion. We have now included a supplemental figure (Figure S3), showing the dose response of pCaMKII bound to different species. We have also added the following sentence to the last paragraph of page 7 of the manuscript :

"This observation is true for p-mCaMKII bound to any species of Ca^{2+}/CaM, and, as expected, with increasing amplitude of Ca^{2+} spikes, the largest role is played by CaMs that are bound to 4 Ca^{2+}s (Figure S3)."

3.8. Until Figure 4, the authors emphasize that there exists a calmodulin concentration ($\sim 30\mu M$) for which the presence of Ng favors CaMKII phosphorylation. However, this facilitation seems to be gone when simulation calcium transients (Fig. 5-9). What is the reason for this?

From our simulations it appears that, while in principle, the presence of Ng can favor CaMKII phosphorylation at higher $[Ca^{2+}]$s, the 100ms calcium transients are too short to bring this effect to light.

3.9. pg. 14. 1st sentence : We next investigated ... . Which approach was used for the results until that point? What are the differences in the approaches and how to interpret the results from both? Why are the results quantitatively different in terms of peak phosphorylated CaMKII concentration, for example (Fig. 6)

We apologize for any confusion our wording might have caused. Until this point in the manuscript, we used a constant 10 µM calcium throughout the simulations, and we switched to $\sim$ 100ms calcium transients at this point to better mimic Ca^{2+} signals occurring in dendritic spines. We have now added the phrase "a constant calcium concentration of $[Ca^{2+}] = 10$ µM" to the first paragraph of the Results section to make it more clear, and extensively edited the manuscript to clarify the differences between the models, conditions and stimuli presented.

3.10. pg. 14. 1st par, line 12 : It is not clear to me why some of the simulations would not yield a change in CaMKII phosphorylation level and other yield a considerable increase in pCaMKII. Can the authors elaborate and explain?

Since the number of molecules involved in this simulations is relatively small, and the simulations are stochastic, not all possible reactions are triggered during a given simulation. Depending on the seed used in a given trial, some of the simulations yield some CaMKII phsophorylation, while other don't. We have now added the following text to the explanation in the third paragraph of page 15 of the manuscript to make it more clear:

"We note that $[CaM] = 30$ µM corresponds to 283 CaM molecules in our stochastic model. Only a fraction of these molecules binds calcium during the calcium transient, and only a fraction of these complexes bind a hCaMKII subunit. Furthermore, only a fraction of these hCaMKII subunits have an active neighbor that can phsophorylate them. Thus, hCaMKII would not always react to the 10 µM free calcium spike, and sometimes there will be no detected phosphorylated hCaMKII subunits. These events were not taken into account in the calculations shown in Figures 6B and 7B, D, F and H."

Minor comment :

Fig. 2 A-D : I would suggest the same x-scale for all four panels. Same for Fig. 3A-C.

We thank the reviewer for this suggestion, we have now modified the figures to have the same x-scales.

Have all data underlying the figures and results presented in the manuscript been provided?

Large-scale datasets should be made available via a public repository as described in the PLOS Computational Biology data availability policy, and numerical data that underlies graphs or

summary statistics should be provided in spreadsheet form as supporting information.

Reviewer 1: Yes

Reviewer 2: Yes

Reviewer 4: Yes
