# Supplementary material for: Interactions between calmodulin and neurogranin govern the dynamics of CaMKII as a leaky integrator
Source: PLoS Comput Biol. 2020 Jul 17;16(7):e1008015. doi: 10.1371/journal.pcbi.1008015 (PMC7390456; doi:10.1371/journal.pcbi.1008015)
Supplement: Attachment — Submitted filename: Ordyan_PLoSCompBiol_R1.pdf [file pcbi.1008015.s007.pdf]

Interactions between calmodulin and neurogranin govern the dynamics of CaMKII as leaky integrator

Mariam Ordyan, Tom Bartol, Mary Kennedy, Padmini Rangamani, and Terrence Sejnowski

In this manuscript, the authors investigate the phosphorylation dynamics of CaMKII triggered through binding of calcium-associated calmodulin. The authors account for the sequestering of calmodulin through neurogranin and show that the presence of neurogranin has a profound impact on available calmodulin and consequently on CaMKII phosphorylation levels and dynamics. The results suggest neurogranin to be potentially important for regulating processes involved in synaptic long-term potentiation. The authors are the first to consider the interaction between calmodulin and neurogranin and the impact for CaMKII activation.
We thank the reviewer for their detailed and constructive suggestions. These suggestions have helped us significantly improve the manuscript. We address their comments and the corresponding changes we made to the manuscript below.

My general critic is that the study falls short in providing the insights and explanations which are the unique potential of such modeling studies. Another crucial part of biophysical modeling work is to propose feasible experiments which can support or contradict the conclusions drawn from the theoretical results and further elucidate the role of the protein interaction network. Such considerations are completely missing in the present work.
We thank the reviewer for this feedback. In the extensively revised manuscript, we have now made major changes to the introduction and the discussion, in addition to providing clarity in the results section throughout the manuscript. We have also discussed the role of the CaM-Ng interaction in the context of other pathways (for e.g. PKC) and how this will impact downstream signaling and structural plasticity. We hope the reviewer finds the revised manuscript is now suitable for publication.

The authors improved reader comprehensions by adding explanatory statements throughout the manuscript. Nevertheless, I would like to follow up on some of my comments and concerns which I felt were not addressed adequately in the manuscript.

I have a couple of main comments and concerns :

1. It's also not clear how the steady-state results, which reveal the “non-intuitive” dependence of pCaMKII on calmodulin concentration (e.g. Fig. 4), relate to the dynamic stimulation part, in which the authors use calcium transients to induce CaMKII phosphorylation (starting with Fig. 5). The facilitating effect of neurogranin in the steady-state results seems to be absent in the dynamic calcium simulations, where the sole effect of Ng appears to be reducing pCaMKII levels. Putting the presented results in relation would help reader comprehension.
We thank the reviewer for this comment. We have now extensively edited to manuscript to clarify the relationship between the different stimuli and different models. Indeed, as the reviewer points out, in the cases when calcium spikes are investigated the facilitating effect of Ng seems to be absent at all [CaM]s. However, our steady state interactions give insight into the relative distributions of calcium-bound CaMs at different levels of [CaM]s setting the stage for a more complete understanding of the role played by Ng in this pathway. Since our goal was to build a bottom-up approach in this paper, we believe that it is important to understand the relevant behavior of our model at every step.

I agree that the steady-state investigations are a useful first step in providing firsts insights into the model behavior. However, the differential effect of Ng in the steady-state behavior is emphasized in the steady-state part of the results (Fig. 4) and completely ignored in the latter part of the manuscript. I still don't understand how to reconcile the two steady-state with the dynamics part.

2. As the authors point out, calmodulin contains four calcium binding sites, two at the C- and two at the N-terminal domain. One hallmark of a steep calcium-dependent activation of calmodulin is that calcium binding happens in a cooperative manner in each one of these pairs (Chin D, Means A (2000) Calmodulin: a prototypical calcium sensor. Trends Cell Biol 10: 322–328). It's surprising that the authors don't account for this property crucially determining how calcium activation affects downstream targets such as CaMKII. The cooperativity strongly favors CaM+2Ca and CaM-4Ca alternating the relative concentrations curves in Fig. 2 and all subsequent results.

We thank the reviewer for this comment. We would like to clarify that cooperativity is built into our model rather than being included explicitly and is reflected in the rate constants presented in Table 1 as demonstrated on the figure below.

OK.

3. Previous work on CaMKII activation through CaM/Ca and autophosphorylation revealed that the protein can exhibit bistability in its phosphorylation level (Zhabotinsky 2000 Biophys J; Graupner 2007 PLoS Comp Biol). In other words, for the same calcium concentration, the protein can exist in a highly or a weakly phosphorylated state and both are stable. How does this current work relate to this line of work. Does CaMKII bistability exist in the model?

We thank the reviewer for this question. We would like to make the following clarications about the notion of CaMKII bistability as presented in the Zhabotinsky 2000 model and point out the following sentence from Zhabotinsky 2000 Biophys J

"Fig. 3 demonstrates that e_K must exceed 10 μ M, and K_M must be signicantly lower than 1 μ M to obtain a bistability range that includes the resting value of the intracellular Ca^{2+} and is wide enough to prevent induction of LTP by random uctuations of Ca^{2+} concentration."

Here e_K refers to [CaMKII], and K_M refers to the Michaelis constant of PP1. The major difference between our model and the one presented in Zhabotinsky 2000 is the source of kinetic parameters. In our model, we used an experimentally measured value of 11 μ M for K_M (Table 1, Bradshaw et al 2003, PNAS) and therefore, we would not expect to see bistability. We have now added a new supplementary figure (Figure S4) and the following paragraph to our manuscript (page 23 last paragraph) to provide better context for our model in light of the bistability argument.

I understand that the model parameters are outside the range where CaMKII would exhibit bistable phosphorylation behavior. However, the presentation of the authors suggest that the difference between previous models and their findings is the “nonlinear rate functions used in the model” (pg. 4, 1st line) and the fact that they use a “more complete computational model of CaMKII dynamics [accounting] for both the behavior of the monomer and the dynamics of CaMKII holoenzyme”. Both are not differences to previous studies mentioned above, which resolve the nature of the inter-subunit phosphorylation in the holoenzyme. Also, the nonlinear rate functions emerge from the non-linear calcium-dependent activation of CaMKII phosphorylation and dephosphorylation (often described by Hill functions). I suspect that the same behavior exists in the presented model?

4. What is the rational behind depicting relative concentration for ca-bound calmodulin in Fig.

2?

Calcium and Ng compete for CaM. By demonstrating how the number of calcium ions bound to CaM in the presence and absence of Ng alters the relative concentration of calcium-bound CaM, we set the stage for understanding how mCaMKII phosphorylation is affected by the presence or absence of Ng. We hope that it can be appreciated that the results depicted on Figure 2 help understand results presented later in the paper (in particular on Figures 3 and 4). Since we have adopted a bottom-up approach in this work we think it important to be able to understand the mechanisms at work at every level of building of the model presented here.

OK.

5. Fig. 3 and Fig. 4 : Why is the concentration of calcium-bound calmodulin bound to phosporylated CaMKII (Fig. 3) and phosphorylated CaMKII (Fig. 4) decreasing with increasing calmodulin concentration? Even though the *relative* free calcium-bound calmodulin decreases with more CaM, the absolute concentration of calcium-bound calmodulin should increase or saturate. I would expect monotonously increasing concentration levels in Fig. 3 and 4.

As the reviewer mentions the relative calcium-bound calmodulin concentration decreases with more CaM. Since calcium-free CaM can still bind CaMKII, albeit with a low affinity, at higher relative concentration these species present a serious competition with underrepresented forms of calcium-bound CaM, eventually out-competing these forms at ultra-high concentrations, and resulting in lower and lower CaMKII phosphorylation.

OK, I understand now. Does this explanation also appear in the results section?

6. pg. 4, 3rd paragraph : What is at the origin of the cross-over of the of the calcium-bound calmodulin with multiple calcium ions between curves in the presence and absence of Ng? Can the authors provide an intuition for this effect which does not exist for calmodulin bound with 2 or 3 calcium ions.

As we explain in the text (now last paragraph on page 5), ... Thus, we conclude that
the relative distribution of different calcium-bound forms of CaM are indeed a result of competition between Ng and Ca^{2+} for CaM."

OK.

7. It would be instructive to see the dynamics of the different calmodulin forms (CaM-1Ca, CaM-2Ca,...) in response to the calcium transient, the summary of which is presented in Fig. 5 for pCaMKII. That could maybe also provide a link to the steady-state considerations discussed up to this point.

We thank the reviewer for this suggestion. We have now included a supplemental

figure (Figure S3), showing the dose response of pCaMKII bound to different species. We have also added the following sentence to the last paragraph of page 7 of the manuscript : \This observation is true for p-mCaMKII bound to any species of Ca2+=CaM, and, as expected, with increasing amplitude of Ca2+ spikes, the largest role is played by CaMs that are bound to 4 Ca2+s (Figure S3)."

OK.

8. Until Figure 4, the authors emphasize that there exists a calmodulin concentration (~30 µM) for which the presence of Ng favors CaMKII phosphorylation. However, this facilitation seems to be gone when simulation calcium transients (Fig. 5-9). What is the reason for this?

From our simulations it appears that, while in principle, the presence of Ng can favor CaMKII phosphorylation at higher [Ca^{2+}]s, the 100ms calcium transients are too short to bring this eect to light.

It is puzzling that even at large peak calcium concentrations (Fig. 5) and repetitions of the calcium transients (Fig. 8 & 9) this effect is absent in the dynamic picture. Do the authors know why? I would wish the authors can make stronger effort in reconciling the steady-state and the dynamical picture messages of the manuscript.

9. pg. 14. 1st sentence : “We next investigated ... “. Which approach was used for the results until that point? What are the differences in the approaches and how to interpret the results from both? Why are the results quantitatively different in terms of peak phosphorylated CaMKII concentration, for example (Fig. 6)?

We apologize for any confusion our wording might have caused. Until this point in the manuscript, we used a constant 10 µ M calcium throughout the simulations, and we switched to 100ms calcium transients at this point to better mimic Ca^{2+} signals occurring in dendritic spines. We have now added the phrase \a constant calcium concentration of [Ca^{2+}] = 10 µ M" to the first paragraph of the Results section to make it more clear, and extensively edited the manuscript to clarify the differences between the models, conditions and stimuli presented.

The additional explanations are clearly helpful to understand the different modeling approaches and conditions used.

10. pg. 14. 1st par, line 12 : It is not clear to me why some of the simulations would not yield a change in CaMKII phosphorylation level and other yield a considerable increase in pCaMKII. Can the authors elaborate and explain?

Since the number of molecules involved in this simulations is relatively small, and the simulations are stochastic, not all possible reactions are triggered during a given simulation. Depending on the seed used in a given trial, some of the simulations yield some CaMKII phosphorylation, while other don't. We have now added the following text to the explanation in the third paragraph of page 15 of the manuscript to make it more clear:

\We note that [CaM] = 30 µM corresponds to 283 CaM molecules in our stochastic model. Only a fraction of these molecules binds calcium during the calcium transient, and only a fraction of these complexes bind a hCaMKII subunit. Furthermore, only a fraction of these hCaMKII subunits have an active neighbor that can phsophorylate them. Thus, hCaMKII would not always react to the 10 µM free calcium spike, and sometimes there will be no detected phosphorylated hCaMKII subunits. These events were not taken into account in the

calculations shown in Figures 6B and 7B, D, F and H."

I understand that the origin of these fluctuations is the stochastic nature of the simulations and the low number of molecules involved. However, why are the outcomes with zero phosphorylation not taken into account ("The events with no detected hCaMKII phosphorylation were not taken into account ... ." pg. 15, 3rd par.) ? Zero phosphorylation is a valid result and should reduce the mean since it reflects that the transition probability of a given reaction is low and therfore does not take place.

Minor comment :

Fig. 2 A-D : I would suggest the same x-scale for all four panels. Same for Fig. 3A-C.
We thank the reviewer for this suggestion, we have now modified the figures to have the same x-scales.
OK.

Fig. 2 and 3 : Which lines represent the simulations with and without Ng ? Labels are missing.
