# Supplementary material for: Interactions between calmodulin and neurogranin govern the dynamics of CaMKII as a leaky integrator
Source: PLoS Comput Biol. 2020 Jul 17;16(7):e1008015. doi: 10.1371/journal.pcbi.1008015 (PMC7390456; doi:10.1371/journal.pcbi.1008015)
Supplement: Attachment — Submitted filename: addressing_comments.pdf [file pcbi.1008015.s008.pdf]

Addressing reviewers comments

We thank the reviewer for their further comments. Below are our responses to their comments in green, and the changes we have made to the manuscript in red.

Reviewer

1. It's also not clear how the steady-state results, which reveal the non-intuitive dependence of pCaMKII on calmodulin concentration (e.g. Fig. 4), relate to the dynamic stimulation part, in which the authors use calcium transients to induce CaMKII phosphorylation (starting with Fig. 5). The facilitating effect of neurogranin in the steady-state results seems to be absent in the dynamic calcium simulations, where the sole effect of Ng appears to be reducing pCaMKII levels. Putting the presented results in relation would help reader comprehension.

We thank the reviewer for this comment. We have now extensively edited to manuscript to clarify the relationship between the different stimuli and different models. Indeed, as the reviewer points out, in the cases when calcium spikes are investigated the facilitating effect of Ng seems to be absent at all [CaM]s. However, our steady state interactions give insight into the relative distributions of calcium-bound CaMs at different levels of [CaM]s setting the stage for a more complete understanding of the role played by Ng in this pathway. Since our goal was to build a bottom-up approach in this paper, we believe that it is important to understand the relevant behavior of our model at every step.

I agree that the steady-state investigations are a useful first step in providing firsts insights into the model behavior. However, the differential effect of Ng in the steady-state behavior is emphasized in the steady-state part of the results (Fig. 4) and completely ignored in the latter part of the manuscript. I still don't understand how to reconcile the two steady-state with the dynamics part.

As the reviewer points out the facilitating effect of Ng observed at constant high $[Ca^{2+}]$ is not present in CaMKII phosphorylation in response to even very large Ca^{2+} pulses. We think that this difference in the behaviour emphasizes the importance of the temporal dynamics of the system.

We have added the following sentence to the first paragraph on page 8 to address this:

This observation, counter-intuitive as it might seem, highlights the importance of the temporal dynamics of the reactions, and demonstrates that drawing conclusions about the system based solely on steady-state considerations alone can be misleading.

3. Previous work on CaMKII activation through CaM/Ca and autophosphorylation revealed that the protein can exhibit bistability in its phosphorylation level (Zhabotinsky 2000 Biophys J; Graupner 2007 PLoS Comp Biol). In other words, for the same calcium concentration, the protein can exist in a highly or a weakly

phosphorylated state and both are stable. How does this current work relate to this line of work. Does CaMKII bistability exist in the model?

We thank the reviewer for this question. We would like to make the following clarifications about the notion of CaMKII bistability as presented in the Zhabotinsky 2000 model and point out the following sentence from Zhabotinsky 2000 Biophys J "Fig. 3 demonstrates that e_k must exceed 10 µM, and K_M must be significantly lower than 1 µM to obtain a bistability range that includes the resting value of the intracellular Ca^{2+} and is wide enough to prevent induction of LTP by random fluctuations of Ca^{2+} concentration." Here e_k refers to [CaMKII], and K_M refers to the Michaelis constant of PP1. The major difference between our model and the one presented in Zhabotinsky 2000 is the source of kinetic parameters. In our model, we used an experimentally measured value of 11 µM for K_M (Table 1, Bradshaw et al 2003, PNAS) and therefore, we would not expect to see bistability. We have now added a new supplementary figure (Figure S4) and the following paragraph to our manuscript (page 23 last paragraph) to provide better context for our model in light of the bistability argument.

I understand that the model parameters are outside the range where CaMKII would exhibit bistable phosphorylation behavior. However, the presentation of the authors suggest that the difference between previous models and their findings is the nonlinear rate functions used in the model (pg. 4, 1st line) and the fact that they use a more complete computational model of CaMKII dynamics [accounting] for both the behavior of the monomer and the dynamics of CaMKII holoenzyme. Both are not differences to previous studies mentioned above, which resolve the nature of the inter-subunit phosphorylation in the holoenzyme. Also, the nonlinear rate functions emerge from the non-linear calcium-dependent activation of CaMKII phosphorylation and dephosphorylation (often described by Hill functions). I suspect that the same behavior exists in the presented model?

Unfortunately, we were quite puzzled by the reviewer's reference to nonlinear rate functions (pg 4., 1st line). We are not sure what the reviewer is referring to here. However, we seek to clarify that we do not approximate the reaction rates through Hill functions, rather all the rate constants used in our model are constant values and rate equations are linear fluxes, as presented in Table 1. The non-linearity of calcium-dependent activation of CaMKII in our model arises from the cooperativity built into the rate constants and reactions. To prevent any further confusion we have now added a list of the reactions used to our supplemental materials (Table 2). As noted in our previous response, based on the experimentally measured parameters used in our model we do not expect nor observe bistability (Figure 4S).

5. Fig. 3 and Fig. 4 : Why is the concentration of calcium-bound calmodulin bound to phosporylated CaMKII (Fig. 3) and phosphorylated CaMKII (Fig. 4) decreasing with increasing calmodulin concentration? Even though the relative free calcium-bound calmodulin decreases with more CaM, the absolute concentration of calcium-bound calmodulin should increase or saturate. I would expect monotonously increasing concentration levels in Fig. 3 and 4.

As the reviewer mentions the relative calcium-bound calmodulin concentration decreases with more CaM. Since calcium-free CaM can still bind CaMKII, albeit with a low affinity, at higher relative concentration these species present a serious competition with underrepresented forms of calcium-bound CaM, eventually out-competing these forms at ultra-high concentrations, and resulting in lower and lower CaMKII phosphorylation.

OK, I understand now. Does this explanation also appear in the results section?

We have now added the following sentance to the first paragraph on page 7 to shed more light upon this observation.

Finally, at ultra-high [CaM] calcium-free CaM becomes the relevant species, which can still bind CaMKII, albeit with a low affinity (Table 1). Since these species do not allow the phosphorylateion of the bound mCaMKII the phosphorylation levels reach 0 at ultra-high [CaM].

8. Until Figure 4, the authors emphasize that there exists a calmodulin concentration (30 µM) for which the presence of Ng favors CaMKII phosphorylation. However, this facilitation seems to be gone when simulation calcium transients (Fig. 5-9). What is the reason for this?

From our simulations it appears that, while in principle, the presence of Ng can favor CaMKII phosphorylation at higher $[Ca^{2+}]$s, the 100ms calcium transients are too short to bring this effect to light.

It is puzzling that even at large peak calcium concentrations (Fig. 5) and repetitions of the calcium transients (Fig. 8 and 9) this effect is absent in the dynamic picture. Do the authors know why? I would wish the authors can make stronger effort in reconciling the steady-state and the dynamical picture messages of the manuscript.

The facilitating effect of Ng is caused by the increase of 3- and 4-Ca^{2+}-bound CaM species in the presence of Ng. This is not the case for a short ~ 100 ms Ca^{2+} pulse of any magnitude: the concentration of 3- and 4-$Ca2+$-bound CaM species is always higher in the absence of Ng in this case (Figure 3S).

10. pg. 14. 1st par, line 12 : It is not clear to me why some of the simulations would not yield a change in CaMKII phosphorylation level and other yield a considerable increase in pCaMKII. Can the authors elaborate and explain?

Since the number of molecules involved in this simulations is relatively small, and the simulations are stochastic, not all possible reactions are triggered during a given simulation. Depending on the seed used in a given trial, some of the simulations yield some CaMKII phosphorylation, while other don't. We have now added the following text to the explanation in the third paragraph of page 15 of the manuscript to make it more clear:

"We note that [CaM] = 30 µM corresponds to 283 CaM molecules in our stochastic model. Only a fraction of these molecules binds calcium during the calcium transient, and only a fraction of these complexes bind

a hCaMKII subunit. Furthermore, only a fraction of these hCaMKII subunits have an active neighbor that can phsophorylate them. Thus, hCaMKII would not always react to the 10 µM free calcium spike, and sometimes there will be no detected phosphorylated hCaMKII subunits. These events were not taken into account in the calculations shown in Figures 6B and 7B, D, F and H."

I understand that the origin of these fluctuations is the stochastic nature of the simulations and the low number of molecules involved. However, why are the outcomes with zero phosphorylation not taken into account (The events with no detected hCaMKII phosphorylation were not taken into account ... . pg. 15, 3rd par.) ? Zero phosphorylation is a valid result and should reduce the mean since it reflects that the transition probability of a given reaction is low and therfore does not take place.

The reasoning behind this is that we are interested in quantifying an average effect in a spine, whenever CaMKII phosphorylation occurs in response to a signal. Including the zero phosphorylation results in the calculation would underestimate the CaMKII phosphorylation level in the spines that do exhibit CaMKII phosphorylation. In the interest of describing the effect on a single spine level we consider it more informative to quantify the CaMKII phosphorylation when it is present, and report the percentage of the events where it is not.
